# Why validation is not enough: Setting the scene for the implementation of the Kimberley Mum’s Mood Scale

**DOI:** 10.1371/journal.pone.0234346

**Published:** 2020-06-12

**Authors:** Emma Carlin, Erica Spry, David Atkinson, Julia V. Marley

**Affiliations:** 1 The Rural Clinical School of Western Australia, The University of Western Australia, Broome, Western Australia, Australia; 2 Kimberley Aboriginal Medical Services, Broome, Western Australia, Australia; University of Oxford, UNITED KINGDOM

## Abstract

**Background:**

The two part Kimberley Mum’s Mood Scale (KMMS) has been developed and validated as a culturally appropriate perinatal depression and anxiety screening tool for Aboriginal women living in the sparsely populated Kimberley region of North West Australia. As part of implementation aspects of user acceptability were explored to improve clinical utilisation of the KMMS.

**Methods:**

Eighteen health professionals involved in perinatal care participated in an online survey or a qualitative semi-structured interview. Ten Aboriginal women (who held administrative, professional or executive roles) were subsequently interviewed in depth to further explore aspects of KMMS user acceptability.

**Results:**

Many of the health professionals were not using the second part of the KMMS (the psychosocial discussion tool). Time constraints and a perception that the KMMS is only appropriate for women with literacy issues were identified by health professionals as significant barriers to KMMS uptake. In contrast the Aboriginal women interviewed considered the KMMS to be important for literate Aboriginal women and placed high value on having the time and space to ‘yarn’ with health professionals about issues that are important to them.

**Conclusion:**

Implementing the KMMS across the Kimberley region requires health professionals to be trained. It also requires strategic engagement with health services to ensure health professionals and mangers understand the rationale and significance of the KMMS and are engaged in its successful implementation.

## Background

Good perinatal mental health is important for all women, their children and extended families [1–7]. In Australia one in five women are reported to suffer from anxiety or depression in the perinatal period [3], and the reported rate is higher again for Aboriginal women [1, 8–10]. Colonisation, forced removal of Aboriginal people from their cultural homelands and their families, and ongoing policies of discrimination have created conditions for increased stress and mental illness within Aboriginal families [11–13]. These conditions are often further amplified by limited mental health literacy and/or cultural norms governing the concept of mental ill-health, [14, 15] and issues of equity and access to appropriate healthcare providers [16, 17] including mental health specialist services [18].

Routine screening is understood to be an effective clinical strategy in identifying and responding to mental health concerns during the perinatal period [3]. The Australian Government launched the inaugural National Perinatal Depression Initiative (2008–2013) which recommended screening women throughout the perinatal period using the Edinburgh Postnatal Depression Scale (EPDS) [19]. Health professionals in the remote Kimberley region of North West Australia supported the move to formalise screening, but had concerns that the language and concepts used in the EPDS were not appropriate for Kimberley Aboriginal women, and therefore unintentionally disengaged them from the screening process [7].

Following an extensive consultation and co-design period, the Kimberley Mum’s Mood Scale (KMMS) (S1 File) was developed to better address the needs and context of perinatal mental health screening for Kimberley Aboriginal women [7]. The KMMS is a two part tool designed to be verbally administered by the health professional, sitting alongside the woman. Part 1 of the KMMS adapts the EPDS [20] using language and graphics determined via the co-design process. Part 2 involves the conversational approach of ‘yarning’ [21, 22] as a method for health professionals to explore selected psychosocial risk and protective factors with women [23, 24]. Part 1 and Part 2 are interpreted by the health professional to determine the woman’s overall risk of depression and/or anxiety. Results from the Kimberley validation study with 91 women demonstrated that the KMMS is capable of identifying women with moderate or severe risk of depression and/or anxiety when assessed against a blinded reference standard assessment (sensitivity 83%; specificity, 87%; positive predictive value, 68%) [25]. The validation study also demonstrated the KMMS was acceptable to women and their health professionals [25].

The development of the KMMS is part of a broader movement driven by Aboriginal communities, health professionals and researchers to have clinical screening tools that account for and are responsive to the needs and context of Aboriginal patients. This includes studies that are examining the validity of ‘mainstream’ screening tools with Aboriginal populations [26, 27]; adaptation studies which validate an ‘Aboriginal version’ of an existing tool [25, 28, 29]; and the development and validation of new Aboriginal specific screening tools [30–32]. These and other emerging endeavours [33] inform our understanding of what acceptable clinical screening practises look like for Aboriginal Australians.

Several studies detailing the development and validation of Aboriginal specific screening tools have been published [17, 19, 21–25], few studies however, report on the process and outcomes of clinical implementation [34, 35]. Without this lens of enquiry it is impossible to determine if the overall objective for developing Aboriginal specific screening tools, namely health equity, is achieved. The Dynamic Sustainability Framework (DSF) [36] is a discrete model within the implementation science discipline [37, 38]. The DSF recognises that to be successfully sustained, an intervention must successfully ‘fit’ within the clinical setting and the broader ‘ecological’ context [36]. Understanding the ‘fit’ of an intervention requires a rigorous assessment of the intervention (in this case the KMMS) for both health professionals and patients.

User acceptability explores and ultimately assesses if and how, recipients or administrators of a healthcare intervention consider it to be appropriate [39, 40]. Constructs in the assessment of user acceptability include: tool content (language, structure, format etc.); process of screening (emphasis on the type/quality of relationship between the administering health professional and the patient) [40]; intervention coherence (understanding the rationale behind the tool); affective attitude/ethicality; and burden/opportunity costs [39]. The DSF provides a structure for understanding and actioning user acceptability feedback and thus enhancing clinical implementation of Aboriginal specific screening tools. The Kimberley region obtained resources to implement, revalidate and evaluate the KMMS in a real-world setting. Using the DSF this paper explores user acceptability of the KMMS through the lens of health professionals and a select group of Aboriginal women to identify and ultimately address barriers that restrict clinical uptake.

## Methods

User acceptability lends itself to a qualitative methodology in which the experiences and perceptions of health professionals, as they relate to the KMMS, are explored [26, 34, 39, 40]. We adopted a methodological approach of qualitative descriptive [41] as this was a real-world quality improvement study intended to identify and address user acceptability concerns to improve implementation. The project needed to engage with the widest number of health professionals possible in an expedient fashion without compromising the richness of the data collected. As such health professionals were given a choice to participate in an anonymous 10 question on-line survey or a de-identified semi structured interview (S2 File).

To locate health professionals engaged in the delivery of the KMMS, KMMS training records were reviewed from 2015–2017. This is the time period after the KMMS validation study but before additional resources were obtained for the implementation study. During this time period two maternal child health professionals (Kimberley Aboriginal Medical Services and Western Australian Country Health Services–Kimberley) delivered KMMS training to 89 professionals. Of these, 74 were health professionals and 48 were known to be still working in a Kimberley health service. Using a purposive sampling frame [42] these 48 health professionals were emailed and invited to take part in the survey or an interview to discuss their experiences and perceptions of the KMMS. The research team were not involved in providing KMMS training to these health professionals.

Eighteen of the 48 health professionals (37% response rate) responded: ten chose to participate via the online survey and eight via in-depth interviewing (Sample Frame A: Health professionals). Dependant on the location of the health professional the in-depth interviews either took place face to face in a quiet and confidential space of the participants choosing (n = 6) or via the telephone (n = 2). All interviews from Sample Frame A were undertaken by first author EC. In-depth interviews were audio recorded and later transcribed verbatim.

Data from the transcribed in-depth interviews were descriptively and iteratively coded by members of the project team [43] using NVivo 11 (QSR International). Data from the on-line surveys were then coded according to the codes established during coding of the in-depth interviews with additional codes created as needed. The final pooled coded data was collectively reviewed by the team as we sought to explore patterns and themes. During the thematic analysis workshops we identified a difference in the thickness of data between the in-depth interviews and the online surveys. While this limited our ability to explore select themes in the surveys we note that the surveys did allow us to achieve an increased level of participation from healthcare professionals and to make surface level identification of patterns and themes from those respondents [41, 42, 44]. All quotes used in the results section for Sample Frame A were obtained via the qualitative interviews.

During the analysis it became clear that many health professionals perceived the KMMS was only appropriate for Aboriginal women who had low literacy levels or spoke English as a second language. It was therefore important that we developed a second sample frame of ‘educated’ and ‘literate’ Aboriginal women to explore their perceptions of user acceptability. Using purposive sampling we approached ten Aboriginal women living in Broome who at the time of the study held an administrative, professional or executive role (inclusive of maternity leave status) and invited them to participate in the study. The sampling frame was used as a means to engage ‘educated’ and ‘literate’ women. All ten women (Sample frame B: Professional Kimberley Aboriginal Women) participated in face to face in-depth interviews in which semi structured open-ended questions were utilised to explore constructs of KMMS user acceptability. Interviews were undertaken by authors EC or ES.

A copy of the EPDS and KMMS was given to participants during the interview. All participants provided informed consent prior to participating in the study, this included consent to audio record the interviews. As part of the consenting process the researcher talked to the participants about the sensitive nature of the research questions and that if at any stage the woman felt distressed or upset, the interview would be stopped without any negative consequences. The woman and the researcher talked about relevant support options in the event of a woman becoming upset, noting that formal social and emotional wellbeing support may not be available or desired.

We note that all of the women who participated in the study were known to the researchers, they were either work colleagues or acquaintances but were otherwise separate from the project. They had no involvement in the administration of the KMMS, were not part of the maternal child health workforce and were not involved in research in the Kimberley. The interview data was transcribed verbatim from the audio recordings and a copy of the transcript was provided to all participants. Participants were given two weeks to contact the research team if they wished to omit information from, or add information to, their transcript. No participants chose to adjust their original transcript. The transcribed interviews were then descriptively and iteratively coded [43] by the team, inclusive of an Aboriginal research officer (ES), using NVivo 11 (QSR International). The team was then involved in a series of workshops to thematically analyse the data. The inclusion of the broader team in the coding and analysis was a deliberate strategy to ensure any potential bias in interviewer-interviewee relationships was minimised.

This project was endorsed by the Kimberley Aboriginal Health Planning Forum Research Subcommittee and has approval from the Western Australian Aboriginal Health Ethics Committee (Project 781) and the Western Australian Country Health Human Research Ethics Committee (RGS 206).

### Actioning data results

**Fig 1:**
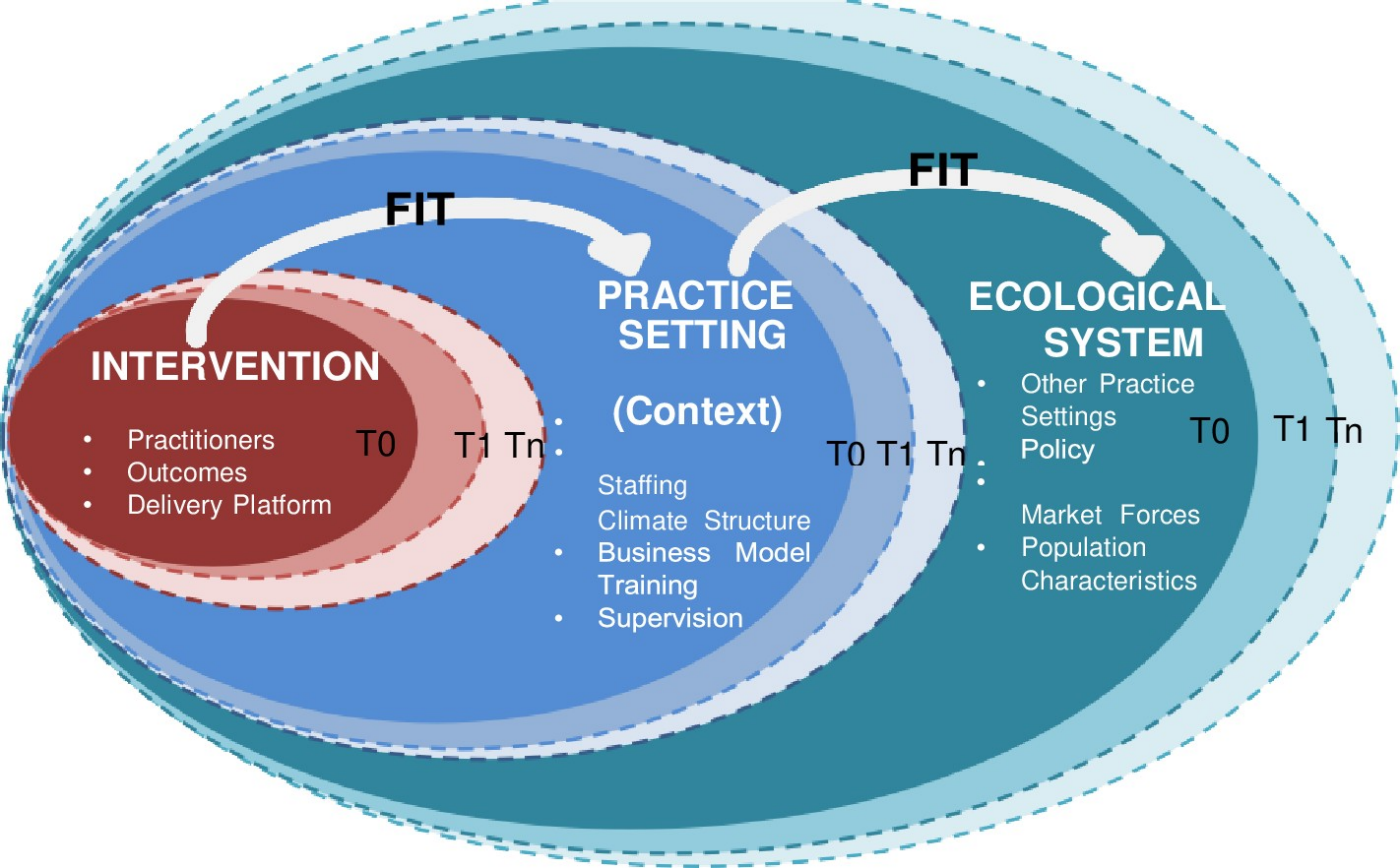
The dynamic sustainability framework. Reprinted from [36] under a CC BY license, with permission from David A. Chambers, original copyright 2013. Maximizing the fit between intervention, practice settings, and the broader ecological system over time (represented by T_0_, T_1_, …, T_n_), each of which has constituent components that may vary [36].

The results from Sample Frame A and Sample Frame B had direct and immediate implications for the next steps of the KMMS implementation project. The findings from Sample Frame A and B have been summarised and in consultation with the project team ranked for implementation significance and then actioned. The implementation results are reported on under the DSF headings of intervention (KMMS tool), practise setting (context) and ecological system [36]. The data from this study has been determined as a T1 assessment (T0 refers to the assessments undertaken at the time of the validation study (Fig 1)).

## Results

### Study participants

#### Sample frame A: Kimberley health professionals

The respondents identified as Midwives [7], Child Health Nurses [5], GP [1], Psychologist [1], and Nurse Manager [1]. Three respondents did not identify their role. On average the respondents had been in the Kimberley for three years and had used the KMMS between five and 10 times and none identified as Aboriginal.

#### Sample frame B: Professional Kimberley Aboriginal women

The women who participated in the study were between the ages of 23–45, they all had children in their immediate care, and all worked in Aboriginal Community Controlled Organisations, including several in Aboriginal Community Controlled Health Services. All participants had grown up in Broome or surrounding areas in the West Kimberley and had long term connections to the Broome Aboriginal community through family and kinship ties.

#### Aboriginal perinatal mental health

Health professionals who participated in the in-depth interviews (n = 8) were asked if they think perinatal mental health is a significant issue for their patients. Most respondents discussed addressing mental health concerns as one of many complicated aspects of supporting Aboriginal women’s overall health and wellbeing. Health professionals identified that ‘trauma’, ‘grief and loss’ and conflict and violence all impacted on Aboriginal women's sense of wellbeing. Half of the health professionals discussed alcohol or drug use (either the person concerned and/or the partner and/or other close family) as contributing to mental health concerns. A paucity of mental health and/or associated social support services was identified by the majority of the health professionals as a major disincentive for engaging women in conversations about their perinatal mental health and social and emotional well-being.

*We see perinatal depression and anxiety but this is a continuum of social disadvantage and intergenerational trauma*. *We have super complicated patients with so many problems*. *Where do we fit mental health in when there are so few resources to respond properly*? Sample frame A, Respondent 13.

When asked about their experiences or perceptions of perinatal mental health concerns, half of the participants in Sample Frame B spoke about their own or a close family member’s experience of perinatal mental ill-health. It was widely agreed that the topic is not often discussed, even between close family members or friends. If it is discussed, it was usually after a lengthy passage of time. ‘Shame’ and the unknown consequences of having and/or disclosing a mental health disorder were identified as significantly contributing to the silence.

*I guess they don’t really show it*. *I know my cousin had a baby like a year ago*, *and she only recently just told me that she had depression after…*. *We didn’t even know*. *A lot of people just hide it I guess*. Sample frame B, Participant 5.

*Well everyone knows that Indigenous people have that big shame factor*, *but I think there is the unknown as well*. *You know if they have postnatal depression what does that mean to them*? *What does it mean for their families or their partners*? *No-one really knows…* Sample frame B, Participant 4.

Participants spoke of how Aboriginal women in the Broome community will talk about feeling ‘not right’, ‘stressed out’ or ‘wild’ and that these terms were often proxies for a range of complicated feelings including depression and anxiety. It was noted that mental health concerns in pregnancy for Aboriginal women do not exist in a vacuum but rather they are situated in the broader life experiences of the woman. Pregnancy was regarded as a time where existing stressors or vulnerability was heightened for women.

#### Perceived appropriateness of the KMMS–Part 1

Health professionals were generally accepting of KMMS Part 1. The language, pictures and style were described as ‘appropriate’ and ‘approachable’ for their patients. Four of the eighteen health professionals however had concerns with the KMMS. These concerns were focussed around a perception that the KMMS is only suitable for women who have low literacy levels.

*Some women will find the use of simple words and pictures insulting*, *especially those with a higher education*. Sample frame A, Respondent 10

The majority of health professionals disclosed using Part 1 of the KMMS without Part 2. Part 1 was seen as ‘quick’, ‘casual’ and ‘friendly’. One health professional suggested validating Part 1 as a ‘stand-alone tool’ stating this would greatly improve the uptake of the KMMS across the region.

Nine out of 10 Aboriginal women from Sample frame B, when asked about the appropriateness of the words and language constructs in Part 1 of the KMMS identified them as appropriate. Participants discussed the simplicity of the language and use of visual aids as the rationale for their answer. The other respondent felt that one question was leading but still felt that overall KMMS Part 1 had better language than the EPDS.

*I think the language is a lot better than the other one*, *whatever it is called*? Sample frame B, Participant 3.

*It’s simple and it’s easy to understand*, *yeah*, *I think that the language is quite good… I reckon that they are the right questions to be asking*. Sample frame B, Participant 10.

*I can’t sleep because I am sad or think too much*. *But it might just be*, *I can’t sleep for another reason*. *Like*, *you’re sort of just putting the answer in my mouth… but yeah*, *that sort of stuff* [pointing to the EPDS question ‘things have been getting on top of me’*]*, *you wouldn’t get a clear answer*. *The Kimberley one will give you clear and more accurate answer*. *Rather than that question*, *because I would probably be like*, *nothing is on top of me*. Sample frame B, Participant 2

All participants in Sample Frame B discussed referring to the words in Part 1 of the KMMS rather than visuals. When asked about visuals, the majority of participants identified them as being useful for women who could not read or had English as a second language.

#### Perceived appropriateness–Part 2

Most health professionals identified value in having a targeted conversation with a woman about her perinatal mental health and wellbeing and expressed that many Aboriginal women faced multiple and complex stressors during the perinatal period. However, Part 2 of the KMMS was often viewed as ‘aspirational’ as opposed to ‘realistic’. Health professionals reported having insufficient time to complete Part 2, primarily due to competing clinical demands.

*I feel the depth of questions is very important but the reality is time constraints when the priority is child health assessments and immunisations*. Sample frame A, Respondent 5.

*Time is a huge constraint if you have broken down the barriers then it is going against everything to rush the conversation*. *As I said before the lack of services and referral options is disheartening …*. *There is no counsellor here for months*. *I ask her to open up but what can I do*? *There is no safe home*, *I can’t make any promises*. *I look to the family and family based supports but I feel I have little choices to offer her*. *Often the family are struggling with their own things–grandmas get old*, *sisters have kids*. *It can be hard sometimes to find family who have space and current capacity*. Sample frame A, Respondent 16.

Two health professionals stated they did not ‘like’ Part 2 and questioned the rationale behind certain domains. These health professionals felt the KMMS positioned them as ‘counsellors’ and put them in a situation where they were exposed to complex information that was often confronting and without immediate resolution. One health professional likened Part 2 to opening up ‘Pandora’s Box’, the other stated it was a ‘waste of time’. These respondents participated via the online survey so it was impossible to further unpack or contextualise these results.

In contrast, health professionals who identified as routinely using Part 2 described the tool as ‘powerful’ and stated the process of completing a KMMS enhanced rapport with patients. These professionals mentioned psychosocial care as part of routine clinical practise and felt there were ‘natural and respected limits’ around the assistance they could provide.

*Generally I think women are keen to share some of their problems with us as nurses even though we cannot solve these issues as such but we can listen*, *we can advise them where to seek help and how we can assist as a support for some of their problems*. Sample frame A, Respondent 9.

Part 2 of the KMMS was highly valued by the Aboriginal women in Sample frame B. They stated the topics in Part 2 encourage a woman ‘to think about her life’ and be viewed ‘holistically’ by her health professional. It was suggested that having a conversation or ‘yarning’ engages women and encourages them to open up. Participants strongly identified that Part 2 could be of high benefit to the social and emotional wellbeing of a woman.

Interviewer 1: *How about if this [KMMS] was given to you at a routine antenatal appointment* [hands the participant a copy of the KMMS Part 1 and Part 2 tool], *would you prefer the EPDS* [hands participant a copy of the EPDS] *were given to you*?

Interviewee: *me*, *I would prefer the mood scale*, *but more the*, *is it*, *part two* [participant located part two and points to it]? *Yeah this one where you are having a conversation that is what I prefer*.

Interviewer 1: Can you tell me why?

*Interviewee*: *I suppose when you are filling out documents like I have done in the past*, *I could easily fake everything that I want*, *just to put up a front*. *But if you are having a general conversation with someone you probably would get more out of them*. *Someone could easily mark that* [Part 1] *and say like ‘oh those are my answers’ but then when you are having a conversation with them it can turn out that it is completely different to what they have put down*. *Having the space to unpack things is really important*. Sample frame B, Participant 5.

#### Cultural safety and delivery of the KMMS (Sample frame B only)

All participants in Sample Frame B identified culturally appropriate tools as important in the delivery of appropriate health care. The women recognised the KMMS as culturally safe. Reasons for this included the simplicity of the language in Part 1 and the yarning component of Part 2.

*When something looks at all of me*, *when it’s holistic like*, *that’s when I know it is culturally ok*. *For our mob health is holistic*, *it looks at social and emotional wellbeing*. *This* [the KMMS] *does that*. *It gets the midwife to think about all of me*, *you know*, *not just my blood pressure and all that*. Sample frame B, Participant 9

One respondent cautioned that while the KMMS looks culturally safe it needs to be delivered in a culturally secure way by trained staff.

*I mean there also has to be that education with who is delivering it as well because anything could look good on paper but again like I said communication*, *relationships*, *all depends on the way you approach it* … Sample frame B, Participant 7.

Relationships, trust and rapport were heavily emphasised in the interviews. Participants spoke about midwives, Aboriginal Health Workers and child health nurses as all having a potential role in administering the KMMS. What was prioritised was the relationship between the woman and her health professional, specifically the woman’s ability to feel comfortable. Confidentiality of information collected during the KMMS was another prominent theme. Participants spoke of how women might be concerned that information they shared during the KMMS could be accessed by child protection services and subsequently ‘used’ to justify removing their child/children. Participants also spoke of concerns with information being ‘shared’ back to the Aboriginal community which could result in relationship problems and family feuding. Women did not identify a preferred health professional to administer the KMMS, instead women identified that having a choice of a trusted Aboriginal health worker or other clinician was important.

*Everyone is different so I think it depends on the person and their relationship*. *They have to feel comfortable with that person*. *Some prefer to have someone they know*, *some people don’t*, *and others would need someone like an Aboriginal Support Worker sitting in there just to explain or to be a presence in the room*, *just to feel safe*. *Yeah*, *as long as they have the option there*, *then they can tell you*, *because everyone will be different*. *The important thing is you feel safe*, *to be able to speak your mind*, *without judgement and knowing that it’s confidential*. Sample frame B, Participant 9.

#### Support for implementation

The majority of health professionals supported implementation of the KMMS across the Kimberley. The KMMS was identified as a valuable ‘approach’ to managing health and wellbeing during the perinatal period. A small group identified the KMMS as a pillar in their delivery of clinical care.

*Absolutely* [support implementation of the KMMS across the Kimberley]. *I believe it is appropriate tool*, *and a vital foundation in identifying risk and opening the dialogue into how it can be explored and managed*. *It is the building block of my relationship with my pregnant women*. Sample frame A, Respondent 7.

Six health professional respondents qualified their support for implementation of the KMMS based on a perception that the KMMS was not appropriate for all Kimberley Aboriginal women. These respondents questioned the use of the KMMS with ‘educated’, or ‘highly literate’ Aboriginal women. Two health professionals did not support implementation of the KMMS into routine clinical practice, citing that the EPDS was sufficient in screening for perinatal depression and anxiety.

*Yes*. *The KMMS for women who need it*, *however we must be sure not to put all women in the same basket as having problem understanding or speaking English* … Sample frame A, Respondent 6.

All Aboriginal women participants from Sample frame B were supportive of the KMMS being introduced across the Kimberley as the primary screening tool for Aboriginal women. They noted that some women might not be comfortable in discussing their stories, and others might want to talk but not have the words for it. Many participants expressed that for some Aboriginal women it could be the first time they had a space to reflect on their life and this could be both confronting and therapeutic. The domains of childhood experiences and relationships were identified as particularly sensitive. Participants warned that these domains should be flagged with sensitivity focusing on the ‘universal’ nature of the questions to put women at ease of being ‘singled out’.

Participants uniformly agreed that it was important for maternal and child health staff to be engaging Aboriginal women in conversations about their social and emotional wellbeing and mental health as part of routine clinical practise. The narrative, holistic approach of the KMMS was identified as the most culturally appropriate way to approach the topic. All participants supported the KMMS as an acceptable feature of their own perinatal care, including two women had received the KMMS as part of their own perinatal care. Notwithstanding the broad support for the KMMS, two participants stated they would not have disclosed a great deal to their health professionals during the KMMS process. They did note that for other women the benefits of yarning may be greater.

#### Actioning data results

The interviews identified a wide range of user acceptability concerns pertinent to the implementation process. These findings are classified under the DSF [26] headings of intervention, practise setting and ecological system. The most critical of these results have been addressed in the results section, the other concerns have been summarised in Table 1 along with the corresponding implementation action identified by the research team.

**Table 1:** Overview of Implementation of the Kimberley Mum’s Mood Scale using the Dynamic Sustainability framework.

**Intervention**			
**Theme**	**Findings**	**Actions**	**Results (as of March 2019)**
Risk assessment	• Steps on how to make a final risk assessment were not clear	• Re analysis of KMMS validation study to identify ‘logic’ behind risk assessment	• Manual revised • Training revised • Presentation at Kimberley Maternal Child Health Forum October 2018 on KMMS risk assessment
Graphics (Part 1)	• Ambiguous (noted by Aboriginal women and health professionals)	• Consulted with staff and Aboriginal women regarding new graphics	• KMMS Graphics updated in Manual
• Alignment to EMR	• Not on ACCHS EMR	• Engaged with MMEx (ACCHS EMR)	• KMMS listed as a clinical item under ANC/MCH care plan • Training revised to ensure health professionals are aware of how to input KMMS data into EMR
**Practice setting (context)**			
**Theme**	**Findings**	**Actions**	**Results (as of April 2019)**
Involvement of Aboriginal staff	• Few Aboriginal staff have been trained as administrators of the KMMS • Health professionals and Aboriginal women see a role for Aboriginal staff	• Discussed findings with clinics	• Six Aboriginal health professionals have been involved in Administrator training across the Kimberley region out of a total of 19 *Note training is ongoing across the Kimberley
Training	• Training not sufficient; whole of clinic training needed and more detailed training for those delivering the KMMS	• Project team re-designed training	• Training offered in two parts: ∘ *Orientation to the KMMS* (50 minutes) designed to be delivered at clinical in-service meeting to all staff. Key areas: overview of perinatal mental health, and development and implementation of the KMMS ∘ *KMMS Administrator training* (1.5 hours) designed to be delivered to staff using the KMMS. Training focuses on the development of the KMMS, it’s acceptability for Aboriginal women, why Part 2 is important, how to complete Part 2, how to ‘assess’ risk, role of clinicians in providing psycho-social support, clinical and non-clinical support pathways
Other clinical constraints	• Time • Other family members present • Competing clinical demands • Quality of the relationship with patient and her health professional (raised by Aboriginal women) • Patients concerns about confidentiality/ sensitivity of KMMS information and patient’s ‘shame’ to engage with KMMS (raised by Aboriginal women and Health professionals)	• Discussed concerns with Project Investigators • Regional advocacy around importance of perinatal wellness and impacts on primary health care engagement (ongoing) • KMMS implementation: monitoring time taken to complete KMMS and how health professionals manage family/children being present at appointments (ongoing)	• KMMS guidelines state that the KMMS should not be completed at first ANC visit (due to time constraints), it is recommend booking an extend follow up appointment with the woman. However the training also identifies that the KMMS can be built on over subsequent routine visits recognising that additional appointments may not always be achievable • Training and manual refined to emphasise the importance of building rapport, using open ended questions, active listening and information for patients around the parameters of confidentiality and next steps
**Ecological System**			
**Theme**	**Findings**	**Actions**	**Results**
Practitioners: values regarding Part 2	• KMMS not appropriate for educated women • Part 1 is good (language and style) • Part 2: • Not our role to be a counsellor • Women value the yarn/builds rapport/can promote engagement during perinatal period • No referral options for follow up • Takes too long	• Interviewed a sample of professional Aboriginal women; they said the KMMS is appropriate for them • Re-analysis of KMMS validation data to demonstrate efficacy of Part 2 in determining risk (ongoing) • Service mapping of key social support services across the Kimberley	• KMMS adopted by the KAHPF as the recommended perinatal depression screening tool for Aboriginal women • Training updated to reflect findings from ‘professional’ Aboriginal women and emphasise the involvement of Aboriginal women in development of the KMMS • Presentation at Kimberley Maternal Child Health Forum October 2018 to discuss findings from Professional Aboriginal women • Training and manual updated: role of health professionals in providing psycho-social support and the importance of Part 2 in the determination of risk • Regional service list / referral options spreadsheet developed and disseminated to health professionals at training
Aboriginal women: values regarding KMMS	• High levels of perinatal mental health concerns amongst family and friends • KMMS language is appropriate • Holistic/culturally secure	• Reporting findings back to health professionals • Refine user acceptability evaluation methodology for KMMS implementation study	• Training and manual revised • Presentation at Kimberley Maternal Child Health Forum October 2018 • User acceptability evaluation methodology submitted to ethics as an amendment

KMMS = Kimberley Mum’s Mood Scale, EMR = Electronic Medical Record, ACCHS = Aboriginal Community Controlled Health Service, MMEX = a type of EMR used by Kimberley ACCHS, ANC = Antenatal Care, MCH = Maternal and Child Health care

## Discussion

Our findings show that despite broad support for the KMMS from both health professionals and Aboriginal women, implementation of the KMMS into clinical care across the Kimberley was ad hoc and inconsistent. This study illustrates the tension between the rationale for developing a culturally secure tool and the approach currently taken by health professionals when administering the tool.

Aboriginal Australians conceptualise health as holistic, encompassing social, emotional, community and cultural dimensions [45]. Aboriginal women involved in the development [7] and validation [25] of the KMMS and this study (Sample Frame B) have suggested that the ‘holistic’ approach of the KMMS, inclusive of having time and space with a health professional to yarn about psychosocial protective and risk factors makes this approach to screening culturally secure. Delivering culturally secure primary health care to Aboriginal patients is linked to health equity [16, 17, 46, 47]. Key characteristics of culturally secure care include trusting relationships between Aboriginal people and their health professionals, Aboriginal people receiving accessible health information and having sufficient time with a health care provider to discuss their health [39]. For Aboriginal women in the perinatal period, culturally secure primary health care is correlated with improved rates of clinical engagement which in turn is associated with enhanced maternal and child health outcomes [48]. Conversely, Aboriginal women with perinatal mental health disorders are reported as having infrequent attendance at routine antenatal appointments [9]. Given the high levels of perinatal mental health disorders for Aboriginal women [1, 8, 10] and continuing adverse health outcomes for Aboriginal women and their babies [48, 49], culturally secure screening is an important clinical component of perinatal care.

Results from health professionals suggest the time taken to administer part 2 of the KMMS (the psychosocial yarn) is a significant barrier. The clinical screening tools that these health professionals are used to using are typically brief and operate in a ‘closed system’ environment [50] where a patient is audited, via an inventory of questions, against known criteria of a disease or disorder. Patients then choose from a set of predefined answers, which are linked to numerical scores. Risk is determined by tallying the numerical scores, with the overall number directly correlating to classification of risk (i.e. high, moderate, low) [41].

Consistent with tool development within the wider population, briefness remains highly valued in the development of Aboriginal specific screening tools [33, 34, 51]. The screening tools have also generally maintained a closed system approach to screening [29, 30, 52–55]. The effectiveness and appropriateness of closed system approaches to assessing risk is questionable for populations with complex and/or diverse needs as these groups have typically been excluded from the population based studies in which the risk criteria was determined from and validated with [50]. Many Aboriginal specific social and emotional wellbeing or mental health screening tools have been designed with (or by) Aboriginal people and recognise Aboriginal specific expressions or antecedents of mental health disorders [54]. However culturally specific psychometric tests have not been completed at a scale that allows for a consistent and standardised determination of Aboriginal specific criteria of depression, anxiety or social and emotional ill health.

The Here and Now Assessment [32] and the KMMS (Part 2) are the only Aboriginal specific screening tools we are aware of that adopt an ‘open’ approach to determining risk. Both tools adopt a yarning approach [21, 22] to foster a patient led narrative of risks and protective factors. These tools rely on the health professional synthesising the information they receive to determine the patients risk profile. This approach to determining risk lends itself to use in a cross cultural context in which population levels of trauma are high [56], and the mainstream criteria of depression and/or anxiety may not be appropriate to a patient’s cultural or situational framework [50]. The process of yarning, engagement and rapport building with a patient while inductively and iteratively building a risk profile is in keeping with approaches of culturally secure provision of care [36–39] and has been identified by women in this and the previous KMMS studies [7, 25] as such.

At a regional level the KMMS has been endorsed by the Kimberley Aboriginal Health Planning Forum as the recommended perinatal screening tool for all Aboriginal women across the region [57], recognising both its clinical validity and high levels of user acceptability. The four year implementation project provides us the resources to refine the KMMS and revise training to improve the intervention coherence [39] and better align the KMMS to health services and health professional’s commitment to delivering culturally appropriate care. An example of this relates to the findings from the health professionals who identified a perception that the KMMS was not appropriate for ‘educated’ Aboriginal women. This belief impacted on health professionals offering the KMMS universally to all Aboriginal women. The findings of this study have been widely disseminated back to health professionals, emphasising that the findings from Sample Frame B were positively dispositioned towards the KMMS and promoting universal KMMS screening amongst perinatal Aboriginal women.

This was a real world study designed to take place quickly so we could identify and action results salient to the implementation of the KMMS. With this lens in mind we attempted to provide accessible and timely ways for busy health professionals to participate in the study, however the two strongest critics of the KMMS responded via the survey and we felt that our understanding of their experiences and perceptions of the KMMS was limited by the static survey format.

With regards to Sample Frame B two limitations require mention, the first is that all participants were sourced from Broome (the largest regional town in the Kimberley and where the project team work and live). The generalisability of these results for other professional Aboriginal women across the Kimberley is unknown. The decision to have a Broome sample was pragmatic and based on time, availability and cost. The findings, however, were consistent with the development of the KMMS [7], which was primarily developed in the East Kimberley, and the validation of the KMMS [17], which included women from 15 communities across the Kimberley.

## Conclusion

The successful implementation of the KMMS into routine clinical care has the potential to be of benefit to Aboriginal women’s perinatal ‘wellness’. We highlight the importance of understanding and addressing the perspectives of both health professionals and the potential recipients when implementing new screening processes. This study also identifies the need for ongoing monitoring and evaluation of user acceptability as an important pillar of sustainable implementation.

## Supporting information

S1 File Kimberley Mum’s Mood Scale Part 1 and 2. (PDF)

S2 File Survey questions and qualitative interview guide. (PDF)

## References

[1] Australian Institute of Health and Welfare. Experience of perinatal depression: data from the 2010 Australian National Infant Feeding Survey. Canberra:: AIHW; 2012.

[2] Buist A, Bilszta J. National Postnatal Depression Program: prevention and early intervention 2001–2005 final report. National screening program. Melbourne; 2005.

[3] Austin M P, Highet N. Mental health care in the perinatal period: Australian Clinical Practice Guideline Melbourne: Centre of Perinatal Excellence; 2017 [updated 3 July 2019. Available from: https://www.cope.org.au/health-professionals/health-professionals-3/review-of-new-perinatal-mental-health-guidelines/.

[4] Hay DF, Pawlby S, Waters CS, Sharp D. Antepartum and postpartum exposure to maternal depression: different effects on different adolescent outcomes. J Child Psychol Psychiatry. 2008;49(10):1079–88. 10.1111/j.1469-7610.2008.01959.x 19017024

[5] Huizink AC, Mulder EJ, Buitelaar JK. Prenatal stress and risk for psychopathology: specific effects or induction of general susceptibility? Psychol Bull. 2004;130(1):115–42. 10.1037/0033-2909.130.1.115 14717652

[6] Stein A, Pearson RM, Goodman SH, Rapa E, Rahman A, McCallum M, et al Effects of perinatal mental disorders on the fetus and child. Lancet. 2014;384(9956):1800–19. 10.1016/S0140-6736(14)61277-0 25455250

[7] Kotz J, Munns A, Marriott R, Marley JV. Perinatal depression and screening among Aboriginal Australians in the Kimberley. Contemp Nurse. 2016;52(1):42–58. 10.1080/10376178.2016.1198710 27294330

[8] Australian Institute of Health and Welfare. The health and welfare of Australia's Aboriginal and Torres Strait Islander Peoples. Canberra: AIHW; 2019.

[9] Gausia K, Thompson S, Nagel T, Rumbold A, Connors C, Matthews V, et al Antenatal emotional wellbeing screening in Aboriginal and Torres Strait Islander primary health care services in Australia. Contemp Nurse. 2013;46(1):73–82. 10.5172/conu.2013.46.1.73 24716765

[10] Prandl KJ, Rooney R, Bishop BJ. Mental health of Australian Aboriginal women during pregnancy: identifying the gaps. Arch Womens Ment Health. 2012;15(3):149–54. 10.1007/s00737-012-0276-0 22476364

[11] Dudgeon P. Close the Gap: psychology. Med J Aust. 2009;190(10):546. 10.5694/j.1326-5377.2009.tb02557.x 19450197

[12] Bowen A, Duncan V, Peacock S, Bowen R, Schwartz L, Campbell D, et al Mood and anxiety problems in perinatal Indigenous women in Australia, New Zealand, Canada, and the United States: A critical review of the literature. Transcultural psychiatry. 2014;51(1):93–111. 10.1177/1363461513501712 24065605

[13] Gausia K, Thompson SC, Nagel T, Schierhout G, Matthews V, Bailie R. Risk of antenatal psychosocial distress in indigenous women and its management at primary health care centres in Australia. Gen Hosp Psychiatry. 2015;37(4):335–9. 10.1016/j.genhosppsych.2015.04.005 25920681

[14] Vicary D, Westerman T. That’s just the way he is’: Some implications of Aboriginal mental health beliefs. Australian e-Journal for the Advancement of Mental Health. 2004;3(3):103–12.

[15] Carlin E, Atkinson D, Marley JV. ‘Having a quiet word’: yarning with Aboriginal women in the Pilbara region of Western Australia about mental health and mental health screening during the perinatal period. Int J Environ Res Public Health. 2019;16(21):4253. 10.3390/ijerph16214253 PMC6862568 31683908

[16] Davy C, Harfield S, McArthur A, Munn Z, Brown A. Access to primary health care services for Indigenous peoples: A framework synthesis. Int J Equity Health. 2016;15(1):163 10.1186/s12939-016-0450-5 27716235 PMC5045584

[17] Gomersall JS, Gibson O, Dwyer J, O'Donnell K, Stephenson M, Carter D, et al What Indigenous Australian clients value about primary health care: a systematic review of qualitative evidence. Aust N Z J Public Health. 2017;41(4):417–23. 10.1111/1753-6405.12687 28712137

[18] McHugh C, Balaratnasingam S, Campbell A, Chapman M. Suicidal ideation and non-fatal deliberate self-harm presentations in the Kimberley from an enhanced police–mental health service notification database. Australasian Psychiatry. 2017;25(1):35–9. 10.1177/1039856216671682 27733662

[19] Australian Government. National Perinatal Depression Initiative (2008–2013). 2008.

[20] Cox JL, Holden JM, Sagovsky R. Detection of postnatal depression. Development of the 10-item Edinburgh Postnatal Depression Scale. Br J Psychiatry. 1987;150:782–6. 10.1192/bjp.150.6.782 3651732

[21] Bessarab D, Ng'andu B. Yarning about yarning as a legitimate method in Indigenous research. Int J Crit Indig Stud. 2010;3(1):13.

[22] Lin I, Green C, Bessarab D. 'Yarn with me': applying clinical yarning to improve clinician-patient communication in Aboriginal health care. Aust J Prim Health. 2016;22(5):377–82. 10.1071/PY16051 28442021

[23] NSW Department of Health. SAFE START Guidelines: Improving mental health outcomes for parents and infants. NSW2009. p. 61.

[24] Austin MP, Lumley J. Antenatal screening for postnatal depression: a systematic review. Acta Psychiatr Scand. 2003;107(1):10–7. 10.1034/j.1600-0447.2003.02024.x 12558536

[25] Marley JV, Kotz J, Engelke C, Williams M, Stephen D, Coutinho S, et al Validity and Acceptability of Kimberley Mum's Mood Scale to Screen for Perinatal Anxiety and Depression in Remote Aboriginal Health Care Settings. PLoS One. 2017;12(1):e0168969 10.1371/journal.pone.0168969 28135275 PMC5279756

[26] Esler DM, Johnston F, Thomas D. The acceptability of a depression screening tool in an urban, Aboriginal community-controlled health service. Aust N Z J Public Health. 2007;31(3):259–63. 10.1111/j.1467-842x.2007.00058.x 17679245

[27] Chan AW, Skeffington P, Reid C, Marriott R. Research protocol for the exploration of experiences of Aboriginal Australian mothers and healthcare professionals when using the Edinburgh Postnatal Depression Scale: a process-oriented validation study using triangulated participatory mixed methods. BMJ Open. 2018;8(10):e022273 10.1136/bmjopen-2018-022273 30287670 PMC6194483

[28] Esler D, Johnston F, Thomas D, Davis B. The validity of a depression screening tool modified for use with Aboriginal and Torres Strait Islander people. Aust N Z J Public Health. 2008;32(4):317–21. 10.1111/j.1753-6405.2008.00247.x 18782392

[29] Garvey G, Beesley VL, Janda M, O'rourke PK, He VY, Hawkes AL, et al Psychometric properties of an A ustralian supportive care needs assessment tool for I ndigenous patients with cancer. Cancer. 2015;121(17):3018–26. 10.1002/cncr.29433 25946658

[30] Thomas A, Cairney S, Gunthorpe W, Paradies Y, Sayers S. Strong Souls: development and validation of a culturally appropriate tool for assessment of social and emotional well-being in Indigenous youth. Aust N Z J Psychiatry. 2010;44(1):40–8. 10.3109/00048670903393589 20073566

[31] LoGiudice D, Smith K, Thomas J, Lautenschlager NT, Almeida OP, Atkinson D, et al Kimberley Indigenous Cognitive Assessment tool (KICA): development of a cognitive assessment tool for older indigenous Australians. Int Psychogeriatr. 2006;18(2):269–80. 10.1017/S1041610205002681 16403247

[32] Janca A, Lyons Z, Balaratnasingam S, Parfitt D, Davison S, Laugharne J. Here and Now Aboriginal Assessment: background, development and preliminary evaluation of a culturally appropriate screening tool. Australas Psychiatry. 2015;23(3):287–92. 10.1177/1039856215584514 25944764

[33] Armstrong EM, Ciccone N, Hersh D, Katzenellebogen J, Coffin J, Thompson S, et al Development of the Aboriginal Communication Assessment After Brain Injury (ACAABI): A screening tool for identifying acquired communication disorders in Aboriginal Australians. Int J Speech Lang Pathol. 2017;19(3):297–308. 10.1080/17549507.2017.1290136 28425776

[34] Thewes B, Davis E, Girgis A, Valery PC, Giam K, Hocking A, et al Routine screening of Indigenous cancer patients' unmet support needs: a qualitative study of patient and clinician attitudes. Int J Equity Health. 2016;15(1):90. 27286811 10.1186/s12939-016-0380-2 PMC4902957

[35] Janca A, Lyons Z, Gaspar J. Here and Now Aboriginal Assessment (HANAA): a follow-up survey of users. Australasian Psychiatry. 2017;25(3):288–9. 10.1177/1039856217700806 28347145

[36] Chambers DA, Glasgow RE, Stange KC. The dynamic sustainability framework: addressing the paradox of sustainment amid ongoing change. Implement Sci. 2013;8(1):117. 24088228 10.1186/1748-5908-8-117 PMC3852739

[37] Damschroder LJ, Aron DC, Keith RE, Kirsh SR, Alexander JA, Lowery JC. Fostering implementation of health services research findings into practice: a consolidated framework for advancing implementation science. Implement Sci. 2009;4:50 10.1186/1748-5908-4-50 19664226 PMC2736161

[38] Pinnock H, Barwick M, Carpenter CR, Eldridge S, Grandes G, Griffiths CJ, et al Standards for Reporting Implementation Studies (StaRI) Statement. BMJ. 2017;356:i6795 10.1136/bmj.i6795 28264797 PMC5421438

[39] Sekhon M, Cartwright M, Francis JJ. Acceptability of healthcare interventions: an overview of reviews and development of a theoretical framework. BMC Health Serv Res. 2017;17(1):88 10.1186/s12913-017-2031-8 28126032 PMC5267473

[40] El-Den S, O'Reilly CL, Chen TF. A systematic review on the acceptability of perinatal depression screening. J Affect Disord. 2015;188:284–303. 10.1016/j.jad.2015.06.015 26386439

[41] Willis DG, Sullivan-Bolyai S, Knafl K, Cohen MZ. Distinguishing features and similarities between descriptive phenomenological and qualitative description research. West J Nurs Res. 2016;38(9):1185–204. 10.1177/0193945916645499 27106878

[42] Parahoo K. Nursing research: principles, process and issues: Macmillan International Higher Education; 2014.

[43] Srivastava P, Hopwood N. A Practical Iterative Framework for Qualitative Data Analysis. Int J Qual Methods. 2009;8(1):76–84.

[44] Bradshaw C, Atkinson S, Doody O. Employing a Qualitative Description Approach in Health Care Research. Global Qualitative Nursing Research. 2017;4:2333393617742282 10.1177/2333393617742282 29204457 PMC5703087

[45] NAACHO. Defintions of Health: National Aboriginal Controlled Health Services; 2019 [https://www.naccho.org.au/about/aboriginal-health/definitions/].

[46] Government A. Implementation Plan for the National Aboriginal and Torres Strait Islander Health Plan 2013–2023. 2013.

[47] Freeman T, Edwards T, Baum F, Lawless A, Jolley G, Javanparast S, et al Cultural respect strategies in Australian Aboriginal primary health care services: beyond education and training of practitioners. Aust N Z J Public Health. 2014;38(4):355–61. 10.1111/1753-6405.12231 25091076

[48] Jongen C, McCalman J, Bainbridge R, Tsey K. Aboriginal and Torres Strait Islander maternal and child health and wellbeing: a systematic search of programs and services in Australian primary health care settings. BMC Pregnancy Childbirth. 2014;14(1):251. 25073873 10.1186/1471-2393-14-251 PMC4261787

[49] Gracey M, King M. Indigenous health part 1: determinants and disease patterns. Lancet. 2009;374(9683):65–75. 10.1016/S0140-6736(09)60914-4 19577695

[50] Webb L. Tools for the job: why relying on risk assessment tools is still a risky business. J Psychiatr Ment Health Nurs. 2012;19(2):132–9. 10.1111/j.1365-2850.2011.01762.x 22070462

[51] Dingwall KM, Lindeman MA, Cairney S. “You’ve got to make it relevant”: barriers and ways forward for assessing cognition in Aboriginal clients. BMC Psychology. 2014;2(1):13.

[52] Schlesinger CM, Ober C, McCarthy MM, Watson JD, Seinen A. The development and validation of the Indigenous Risk Impact Screen (IRIS): a 13-item screening instrument for alcohol and drug and mental health risk. Drug Alcohol Rev. 2007;26(2):109–17. 10.1080/09595230601146611 17364845

[53] Stephens A, Bohanna I, Graham D, Clough AR. Screening and assessment instruments for use in Indigenous-specific alcohol and drug treatment rehabilitation. J Tropical Psychol. 2013;3:e2.

[54] Le Grande M, Ski CF, Thompson DR, Scuffham P, Kularatna S, Jackson AC, et al Social and emotional wellbeing assessment instruments for use with Indigenous Australians: A critical review. Soc Sci Med. 2017;187:164–73. 10.1016/j.socscimed.2017.06.046 28689090

[55] Haswell MR, Kavanagh D, Tsey K, Reilly L, Cadet-James Y, Laliberte A, et al Psychometric validation of the Growth and Empowerment Measure (GEM) applied with Indigenous Australians. Aust N Z J Psychiatry. 2010;44(9):791–9. 10.3109/00048674.2010.482919 20815665

[56] Atkinson J. Trauma trails, recreating song lines: The transgenerational effects of trauma in Indigenous Australia: Spinifex Press; 2002.

[57] Kimberley Aboriginal Health Planning Forum. Perinatal Depression and Anxiety Protocol https://kahpf.org.au/s/Perinatal-Depression-and-Anxiety.pdf2019 [01/03/2019].

