# Supplementary material for: Why validation is not enough: Setting the scene for the implementation of the Kimberley Mum’s Mood Scale
Source: PLoS One. 2020 Jun 12;15(6):e0234346. doi: 10.1371/journal.pone.0234346 (PMC7292413; doi:10.1371/journal.pone.0234346)
Supplement: S1 File — (PDF) [file pone.0234346.s001.pdf]

Kimberley Mum's Mood Scale (KMMS) Part 1

Think about the past 7 days, not just how you feel today.

NAME:

DOB:

DATE:

	Question				
1.	I can sit down and have a good laugh	Yes, always	Yes, sometimes	No, not much	No, never
2.	I look forward for good things to happen	Yes, always	Yes, sometimes	No, not much	No, never
3.	I blame myself when things go wrong	Yes, always	Yes, sometimes	No, not much	No, never
4.	I worry too much and don't know why	Yes, always	Yes, sometimes	No, not much	No, never
5.	I feel frightened and shaky a lot	Yes, always	Yes, sometimes	No, not much	No, never
6.	I can't handle all the stress or I stress out	Yes, always	Yes, sometimes	No, not much	No, never
7.	I feel really no good, like no-one loves me	Yes, always	Yes, sometimes	No, not much	No, never
8.	I can't sleep because I am sad or think too much	Yes, always	Yes, sometimes	No, not much	No, never
9.	I am so sad I have been crying	Yes, always	Yes, sometimes	No, not much	No, never
10.	I think about doing something bad to myself or others	Yes, always	Yes, sometimes	No, not much	No, never

Kimberley Mum's Mood Scale Part 2

PART 1 SCORE:
NAME:
DOB:
DATE:

Refer to the KMMS Manual for information on how to facilitate the yarn, guidelines for administration, examples of enquiring/exploring questions for each psychosocial domain, how to determine overall risk, and options for follow-up actions.

EXPLORE THE WOMAN'S STORY, NOTING THE SITUATION AND HER PROTECTIVE AND RISK FACTORS

SUPPORT:

MAJOR STRESSORS:

SELF-ESTEEM / ANXIETY:

RELATIONSHIPS:

CHILDHOOD EXPERIENCES:

SUBSTANCE MISUSE:

SOCIAL, EMOTIONAL AND CULTURAL WELLBEING:

OVERALL RISK OF DEPRESSION AND/OR ANXIETY (PLEASE TICK)

Consider Part 1 score and the risk and protective factors identified during Part 2 in determining overall risk

LOW	Self-care recommended	
MODERATE	Clinical assessment within 1 week	
HIGH	Clinical assessment required within 48–72 hours	
IMMEDIATE CONCERNS	Clinical handover required immediately	

FOLLOW-UP ACTIONS: Explore relevant referrals, or next steps with the woman.
