# Supplementary material for: Why validation is not enough: Setting the scene for the implementation of the Kimberley Mum’s Mood Scale
Source: PLoS One. 2020 Jun 12;15(6):e0234346. doi: 10.1371/journal.pone.0234346 (PMC7292413; doi:10.1371/journal.pone.0234346)
Supplement: S2 File — (PDF) [file pone.0234346.s002.pdf]

Survey Questions and Qualitative Interview guide

**Theme**	**Sample Frame A (Health professionals) Survey**	**Sample Frame A (Health professionals) Depth Interview Schedule**	**Sample Frame B (Aboriginal women) Depth Interview Schedule**
Perceptions of postnatal depression and anxiety/ perceptions of prevelance		Do you feel perinatal mental health is a significant issue for your patients?	What do these terms mean to you?<br><br>How do you talk about depression and anxiety in your family community?<br><br>What triggers depression and/or anxiety?<br><br>What supports people to have good wellbeing?<br><br>Have you experienced perinatal depression and/or anxiety or know someone who has?
KMMS use	Do you use the KMMS to screen for depression and anxiety during the perinatal period?<br><br>How many times have you used the KMMS 0-1010-2020 +	Do you use the KMMS to screen for depression and anxiety during the perinatal period?<br><br>How many times have you used the KMMS;<br><br>Are you using Part 1 and Part 2?	Have you received the KMMS in your own perinatal care?
Efficacy and appropriateness of perinatal depression and anxiety screening tools	Are there times when you have used the Edinburgh Perinatal Depression Scale instead of the KMMS? Can you explain the reasons why?<br><br>What are the barriers and enablers to using the KMMS with women?	Are there times when you have used the Edinburgh Perinatal Depression Screening tool instead of the KMMS? Can you explain the reasons why?<br><br>What are the barriers and enablers to using the KMMS with women?	Looking at part one of the KMMS. Can you tell me about your thoughts on the tool?<br><br>Do the questions make sense to you / are they appropriate<br><br>Can you tell me about the language in the screening tool, is it right?

			Can you tell me about the use of pictures/ graphics in Part 1.<br>Can you talk to me about the different domains listed in Part 2.<br>The last bit of the KMMS is about deciding on a woman's follow up actions or next steps- what types of support would be important for you or the women you know?<br><br>Having a look at the EPDS what are some of your views about that screening tool?<br><br>What would be the key barriers for women, such as yourself, engaging in the KMMS screening process?
Administration of the KMMS	How often do you use the KMMS during the perinatal period?<br><br>Are you using it according to the EPDS schedule, more or less? Please tell me a bit about your answer	How often do you use the KMMS during the perinatal period?<br><br>Are you using it according to the EPDS schedule, more or less? Please tell me a bit about your answer.<br><br>Please describe how you monitor the KMMS support/ follow up actions with the patient?	Who do you think would be the ideal clinician to take you through the KMMS? Why is this position the right one for administering the tool?<br><br>When, where, how often should the KMMS be administered?

Cultural security/ appropriate	In your experience do patients respond well to using the KMMS? Please explain your answer.		Is it important for you to have culturally secure screening tools?<br><br>Do you think the KMMS is a culturally secure tool?<br><br>What makes you say it is or is not culturally secure?
Support for implementation of the KMMS across the region	Are you supportive of the KMMS being implemented as standard practise across the region? Can you explain the reasons for this	Are you supportive of the KMMS being implemented as standard practise across the region? Can you explain the reasons for this?	Do you support the implementation of the KMMS as the preferred screening tool for all Aboriginal women in the Kimberley?
Training		Do you feel confident using the KMMS?<br>What are your training and support recommendations	
KMMS improvements	What improvements or changes would you make to the KMMS or how it is used?	What improvement or changes would you suggest?<br>How can we better improve integration of the KMMS within clinical practise?	What improvements or changes would you make to the KMMS or how it is used?
Staff role/ time in the Kimberley	How long have you been working for a Kimberley based Health Service and what is your current role?	How long have you been working for a Kimberley based Health Service and what is your current role	
